# Plasticization Effect of Bio-Based Plasticizers from Soybean Oil for Tire Tread Rubber

**DOI:** 10.3390/polym12030623

**Published:** 2020-03-09

**Authors:** Haoshu Xu, Tao Fan, Neng Ye, Weidong Wu, Daye Huang, Danling Wang, Zhao Wang, Liqun Zhang

**Affiliations:** 1 State Key Laboratory of Organic-Inorganic Composites, Beijing University of Chemical Technology, Beijing 100029, China; Xhs18301442253@163.com (H.X.); ft18801366634@126.com (T.F.); 2017210199@buct.edu.cn (N.Y.); 13810367675@163.com (W.W.); 2 Zhongce Rubber Group Co., Ltd., Hangzhou 310014, China; wkhuangdaye@163.com (D.H.); yogidan@163.com (D.W.); 3 Key Laboratory of Beijing City for Preparation and Processing of Novel Polymer Materials, Beijing University of Chemical Technology, Beijing 100029, China

**Keywords:** modified soybean oil, plasticization effect, crosslink density, modulus

## Abstract

Modified soybean oil (MSO) is synthesized from soybean oil (SO) and sulfur, aiming to reduce the double bond quantity of SO and avoid harmful effects on the crosslink density and mechanical properties of rubber. MSO modified with different weight percentages of sulfur is then used to plasticize tire tread rubber (TR). It is found that the crosslink density and modulus of MSO- plasticized rubber are significantly improved compared with that of SO-plasticized TR. MSO modified with 6 wt % sulfur (MSO-6%) exhibits the best plasticization effect on TR, thus, the plasticization effect of MSO-6% on TR was further studied by adjusting its additive content. Thereafter, the Mooney viscosity, Payne effect, mechanical property of different amount of MSO-6% plasticized TR are studied to investigate their plasticization effect. At the same additive content of plasticizer, the plasticization effect of MSO-6% and a commonly used aromatic hydrocarbon plasticizer (AO) is compared to determine the potential application of MSO on tire tread rubber. It is found MSO shows similar plasticization effect on TR compared with AO. More important, the aging resistance property and wear resistance property of MSO-6% plasticized rubber are better than those of AO-plasticized rubber. Therefore, MSO-6% is a promising bio-based plasticizer for tire tread rubber.

## 1. Introduction

Plasticizers play a very important role in the rubber industry [1,2], because they can reduce rubber viscosity and thus improve the processing properties and filler dispersion, enhance the elasticity, and reduce processing energy consumption. The global amount of plasticizers used in rubber in 2019 reached some 10.3 million tons, most of which were used in tire tread manufacture.

The commercial plasticizers used in tire industry are generally petroleum-based plasticizers, such as paraffin oil, naphthenic oil and aromatic oil (DAE) [3], which are not sustainable. Moreover, the most used aromatic oil contains a large amount of carcinogenic polycyclic aromatic hydrocarbons [4,5]. The release of these aromatic hydrocarbon oils during the production, use and recycling of tires can cause great harm to human health and serious pollution to the environment [3,6]. Note that, these petroleum-based plasticizer with small molecular weight can volatilize during heat processing procedures, resulting in weak rubber mechanical properties [7]. Considering the lack of global petroleum resources and the fact we are facing the risk of petroleum depletion, it is imperative to find an environmentally-friendly, renewable and harmless plasticizers for tread rubber [8].

Plant oils, such as cashew oil, soybean oil and castor oil, were found to be good plasticizers for different rubbers [9,10,11,12]. Among the plant oils, soybean oil is one of the most productive. The global soybean oil production in 2018 exceeded 56 million tons, of which 15% were used in industrial areas [7,13]. Besides the large production and low cost, soybean oil is a potential candidate that could fill the requirements as a new generation rubber plasticizer [14]. More importantly, soybean oil is a reactive plasticizer for rubber because the double bonds in it could react with the double bonds in the rubber molecular chains during the crosslinking procedure [15]. During the cure processing procedure, soybean oil can graft onto the rubber molecular chain and avoid volatilization compared with the non-reactive plasticizers. In our previous work, soybean oil was used to plasticize ethylene propylene diene monomer (EPDM) rubber, and it exhibited promising applications in EPDM plasticization to replace petroleum-based paraffin oil [14]. However, soybean oil can consume the cross-linking agent [16] and thus harm the crosslinking density and mechanical properties of rubber. Especially for the tire tread rubber, a decrease of crosslinking density will harm 300% modulus that is very important property for tire tread. Though these problems can be solved by reducing the number of double bonds via hydrogenation [17] or epoxidatio [18,19], both which will cause high costs. We also note that epoxidized soybean oil has bad compatibility with most of the non-polar tire tread rubber components. To date, there are few reports about the use of plant oil in plasticization of tire tread rubber.

Therefore, in this work, we reacted sulfur with soybean oil to reduce the amount of double bonds; in the meanwhile, a small amount of double bonds was preserved as functional groups to further graft onto the rubber molecular chain. Three kinds of soybean oil plasticizers with various molecular weights and different numbers of double bonds were synthesized by adjusting the sulfur weight percentage to study their influence on the cross-linking density and mechanical properties of a commonly used tire tread rubber (TR), compared with soybean oil-plasticized rubber and a reference rubber without any plasticizer. These soybean-oil-based plasticizers are promising green plasticizers for tire tread rubbers, which could make a great contribution to the tire industry.

## 2. Materials and Methods

### 2.1. Materials

Soybean oil was purchased from Yihaijiali Jinlongyu Cereals, Oils & Foodstuffs Co., Ltd. (Shanghai, China). NR-3L and SBR-2739 was a gift from Hangzhou Zhongce Rubber Group Co., Ltd. (Hanghzou, China). Sulfur was supplied by Shandong Tianshun Chemical Co., Ltd. (Linyi, China). Carbon black N220 was purchased from Tian-jin Dolphin Carbon Black Development Co., (Tianjin, China). The silica VN-3 was supplied by Evonik Degussa (Frankfurt, Germany). TESPT was purchased from Nanjing Capatue Chemical Co., Ltd. (Nanjing, China). Other chemicals and rubber curing additives were all analytically pure and were purchased from HWRK Chemical (Beijing, China). Aromatic hydrocarbons v-700 (AO) was purchased from Hansen & Rosenthal Chemical Co., Ltd. (Hamburg, Germany).

### 2.2. Preparation of Modified Soybean Oil (MSO)

Soybean oil (100 g) and different quantities of sulfur (3, 6 and 9 g) were added to a three-necked round-bottom flask equipped with a thermometer and a condenser. The raw materials were mixed with mechanical string at the rate of 100 rpm, and heated at 160 °C for 60 min with a N_2_ atmosphere (50 mL/min). The products with different quantities of S of 3, 6 and 9 g were named MSO-3%, MSO-6% and MSO-9%, respectively.

### 2.3. Preparation of Rubber Compounds and Rubber Composites

The formula of the rubber compounds is summarized in Table 1. The compounds were formed by a four-step mixing process. The first step is the plasticization of the rubber matrix in a mixer (HAAKE, Karlsruhe, Germany) at a rotor speed of 45 rpm and initial temperature of 80 °C for 2 min. In the second step, silica and carbon black filler, coupling agent, plasticizer, stearic acid and zinc oxide were added into the mixer using the same rotor speed and the same temperature. In the third step, the temperature was increased to 160 °C, and the rotor speed was adjusted to 35 rpm and maintained for 8 min. After the rubber mixture cooled to room temperature, sulfur and accelerator were added to an open mill and mixed to obtain the rubber compounds.

The rubber compounds were vulcanized in a vulcanizer for the optimum cure time (*t*_90_) at 151 °C to prepare the rubber composites. The tire tread rubbers plasticized with different plasticizers were named as TR-SO, TR-MSO-3%, TR-MSO-6%, TR-MSO-9%, respectively. The rubber without plasticizer was named as TR.

### 2.4. Methods

• Chemical structure Characterization

^1^H-NMR measurements of soybean oil and modified soybean oil were performed on an AV600 400 MHz spectrometer (Bruker, Karlsruhe, Germany). ^1^H chemical shifts were determined by using the CDCl_3_ peak as reference. FTIR spectra of modified soybean oil were obtained with a Tensor 27 spectrometer (Bruker, Karlsruhecity, Germany) with a resolution of 2 cm^−1^.

• Size Exclusion Chromatography

The modified soybean oil was dissolved in THF and run on a Breeze gel permeation chromatography (GPC) instrument (Waters, Milford, MA, USA) to determine its molecular weight and dispersion index.

• Mooney Viscosity

The viscosity measurement of rubber compounds was performed by using a Mooney Viscometer M3810C (Huanfeng, Beijing, China). The rubber compounds (20 g) were preheated in the Mooney viscometer at 100 °C for 1 min and then, the viscosity at 5 min was recorded as Z100 °C (1+4).

• Mechanical Properties

A CMT4104 electronic tensile tester (SANS, Tianjing, China) was used to measure the mechanical properties of all vulcanizates according to ASTM D638 with at a crosshead speed of 500 mm/min. According to ISO/DIS 37–1990 the dumbbell-shaped samples (25 × 6 × 2 mm^3^) were prepared according to ISO/DIS 37–1990.

• Crosslink density

A Bruker AVANCE III 400WB solid-state NMR spectrometer was used to characterize the crosslink density of TR. The sample was packed into a 10 mm diameter NMR tube. The loaded sample was heated in an oven at a set temperature for 15 min, so that the temperature of each area of the sample was the same, and then it was moved to the heating zone of the nuclear magnetic instrument with the same temperature as the oven. The sample was stabilized for 5 min and then scanned at 90 °C.

## 3. Results

### 3.1. Synthesis and Characterization of Soybean-Oil-Based Plasticizer

In the rubber industry, sulfur is used as the curing agent to react with the double bonds of rubber molecules for crosslinking. Here, we used different weight percentages of sulfur (3, 6, and 9 wt %) to react with soybean oil under a N_2_ atmosphere, aiming to obtain soybean-oil-based plasticizers with different molecular weights and quantity of double bonds. The molecular weights of the three synthesized plasticizers are shown in Table 2, as tested by a GPC method. The results showed that with increasing quantity of sulfur, both the number average molecular weight (*M*_n_) and the weight average molecular weight (*M*_w_) of the modified soybean oil increased, indicating that soybean oil can react with sulfur to form soybean oil oligomers. It also means that we can adjust the degree of reaction by controlling the weight percentage of sulfur.

**Figure 1:**
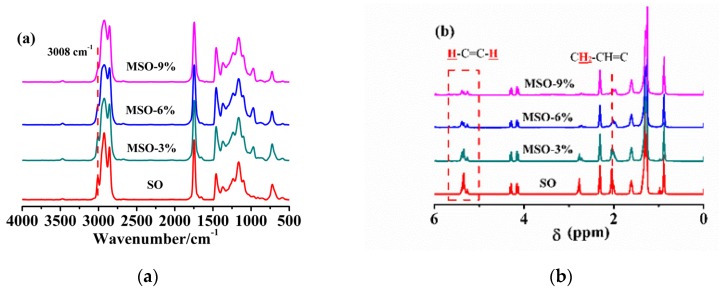
FTIR spectra (**a**) and ^1^H-NMR spectra (**b**) of soybean oil and modified soybean oil with different amounts of reagents.

The remaining quantity of double bonds of the MSO with different sulfur amounts were determined by FTIR (Figure 1a) and ^1^H-NMR (Figure 1b) testing. From the FTIR spectra, the peak at 3008 cm^−1^ represents C=C–H bending vibration of double bonds, and the peak intensity decreased with increasing quantity of sulfur. That is to say, the double bonds of SO were reacted/consumed by sulfur.

In the ^1^H-NMR spectra of SO and MSO, the signal at 5.34 ppm represents the proton of –CH=CH- group, and the signal at 2.02 ppm is attributed to the protons of −CH_2_−CH_2_−CH= group. The intensity of these two signals weakened with the increasing quantity of sulfur, indicating an increased reaction degree. The remaining double bond amounts were calculated from the peak area integrals. Unmodified soybean oil contains an average of 4.6 double bonds per molecule. With the addition of increased amounts of sulfur, the average number of double bonds decreased to 3.4, 2.3 and 1.7, respectively. This statement proved that sulfur can significantly consume double bonds in soybean oil molecules. The GPC, FTIR and ^1^H-NMR results were consistent, which confirmed the reaction between soybean oil and sulfur. The three synthesized plasticizers were then used to plasticize tire tread rubber (TR).

### 3.2. Plasticization Effect of Soybean-Oil-Based Plasticizers with Different Sulfur

#### 3.2.1. Processing Property of the Rubber Compounds

**Figure 2:**
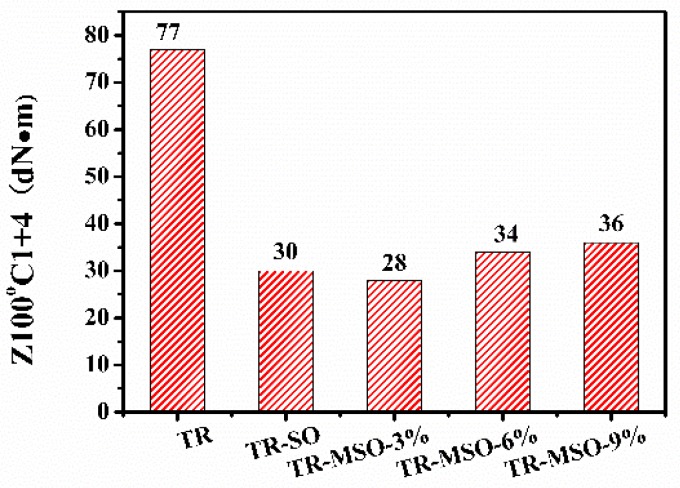
Mooney viscosity of tread rubbers with different plasticizers.

Mooney viscosity testing is the most commonly used method to directly reflect the processing properties of a rubber compound. Low Mooney viscosity is beneficial for good filler dispersion, low energy consumption, and reduced risk of equipment damage. Figure 2 shows the Mooney viscosities of the rubber compounds with no plasticizer or 25 phr plasticizers. It can be seen that the addition of soybean oil or modified soybean oil can significantly reduce the Mooney viscosity of the rubber compounds, indicating they all have a good plasticization effect on the tire tread rubber. With increasing molecular weight, the Mooney viscosity of rubber compound increased slightly. The Mooney viscosities of the compounds plasticized with MSO are similar to that of the soybean oil- plasticized compound.

#### 3.2.2. Curing Behavior

**Figure 3:**
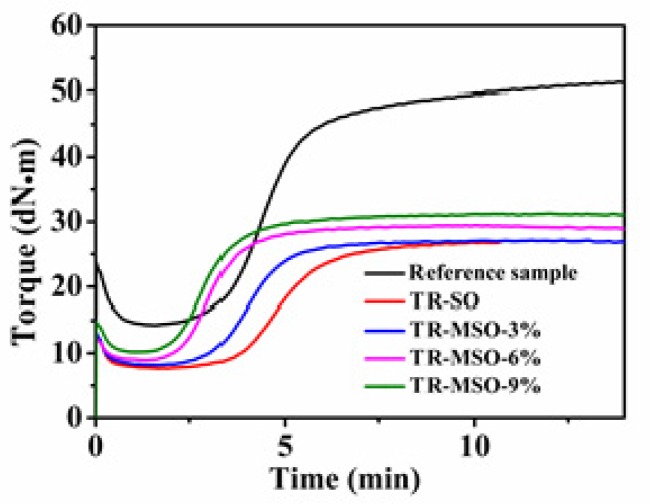
Vulcanization characteristics curve of tread rubber with different plasticizers.

An optimal crosslink density is very important to achieve rubber with good mechanical properties. The addition of plasticizers will generally lead to a decrease in the crosslinking density of rubber [20,21], but as discussed in our previous work, the double bonds of soybean oil would consume crosslinker that results in a lower crosslinking density and weaker rubber mechanical properties. Herein, the curing behavior of the rubber compounds were tested by vulcameter to determine the optimal curing time for preparing the vulcanizates and also compare the crosslinking density from the torque value. Figure 3 shows the vulcanization curves of the rubbers. It can be seen that the torque of the soybean oil- and modified soybean oil-plasticized rubbers are much lower than that of the reference sample, because of the plasticization effect. The torque difference (MH-ML, see Table 3) of TR-MSO is higher than that of TR-SO, and with increasing reaction degree the torque increased. A high torque difference means a high crosslinking density with the same amount of plasticizer. The crosslinking density of TR is also tested by solid state NMR, and the results are shown in Table 3. The crosslinking density of TR-MSO is higher than that of TR-SO, because MSO with a lesser quantity of double bonds did not harm the crosslinking density as SO did. MSO modified with increasing quantities of sulfur led to higher crosslinking density of the TR. We can thus conclude that the influence of soybean oil on crosslinking density could be improved after modification with sulfur. Note that, the T_90_ (the time when the torque reaches 90% of the maximum torque) of TR-MSO is much shorter than that of the SO-plasticized TR. The short T90 means short molding time and high production efficiency, which can also reduce the energy consumption.

#### 3.2.3. Mechanical Property

**Figure 4:**
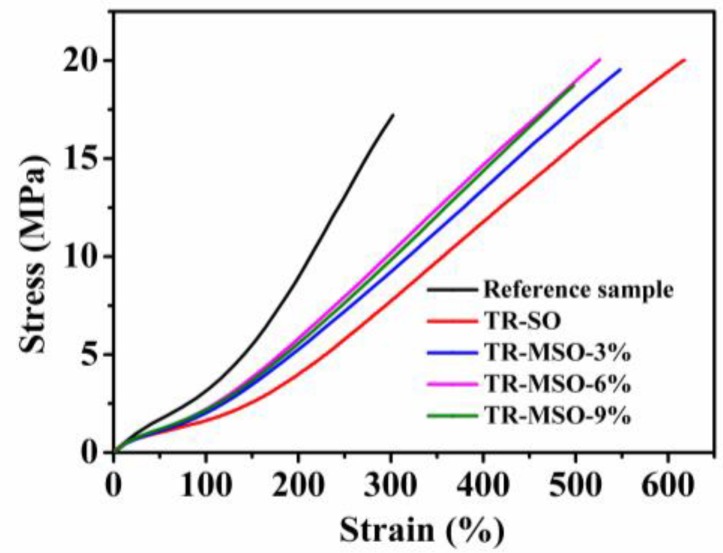
Stress-strain curves of tread rubber with different plasticizers.

The crosslinking density of rubber can influence its mechanical properties, especially the modulus and hardness. It can be seen from Figure 4 and Table 4 that, without plasticizer, the tensile strength of rubber is the smallest because the high viscosity leads to bad processing properties and filler dispersion. Soybean oil-plasticized rubber has good tensile strength, but the 100% modulus and 300% modulus are the lowest, because soybean oil consumed sulfur, which resulted in a small crosslinking density. The lower hardness and higher elongation at break of TR-MSO than TR-SO are also caused by the low crosslinking density. The modulus at 300% of the TR-MSO are higher than that of TR-SO, indicating an improvement of crosslinking density. Note that, the mechanical properties of TR-MSO-6 are the best. Though the number of double bonds in MSO-9 is less than in MSO-6, the tensile strength is lower and the Mooney viscosity is higher due to its high molecular weight and high viscosity. TR-MSO-6% has good processability and it also has the highest modulus at 300%, which is very important for the industrial production of tread rubber. Note that, a high content of sulfur could cause a bad odor that is not welcome in rubber industry. Therefore, different amounts of MSO-6% were used for plasticizing tire tread rubber in order to study the influence of plasticizer amount on the plasticization effect. In order to determine the plasticization effect of MSO-6 on the tire tread rubber, a commercial aromatic hydrocarbons (AO) plasticizer was compared with MSO at the same amount of 25 phr.

### 3.3. Plasticization Effect with Different Quantities of MSO-6% 

#### 3.3.1. Processing Properties

**Figure 5:**
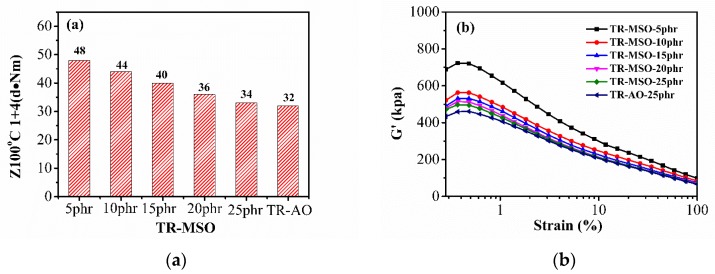
Mooney viscosity (**a**) and G′-strain curves (**b**) of tread rubber with different amounts of plasticizer.

Figure 5a shows the Mooney viscosity of different amounts of TR-MSO-6% compound. When the amount of MSO-6 was increased from 5 to 25 phr, the Mooney viscosity of the resulting ubber compounds decreased continuously. The Mooney viscosity of AO-plasticized TR is slightly lower than that of TR-MSO at the same plasticizer content of 25 phr. The rubber processing analysis (RPA) method was also used to study the processing properties, and the RPA curves are shown in Figure 5b. We can see that with increasing amount of MSO-6S, the initial G’ and the difference between initial G’ and final G’ (ΔG’) became smaller, indicating a low Payne effect and better processing properties for obtaining a better filler dispersion. This phenomenon could be explained by the fact that, with increasing MSO content, the forces between the nanofillers and the rubber molecules are further reduced by the lubrication action. In accordance with the Mooney viscosity result, G’ of TR-MSO-25phr is slightly higher than that of TR-AO. These differences between TR-MSO-25 and TR-AO are not obvious, and could be ignored in the tire industry.

#### 3.3.2. Curing Characteristics

**Figure 6:**
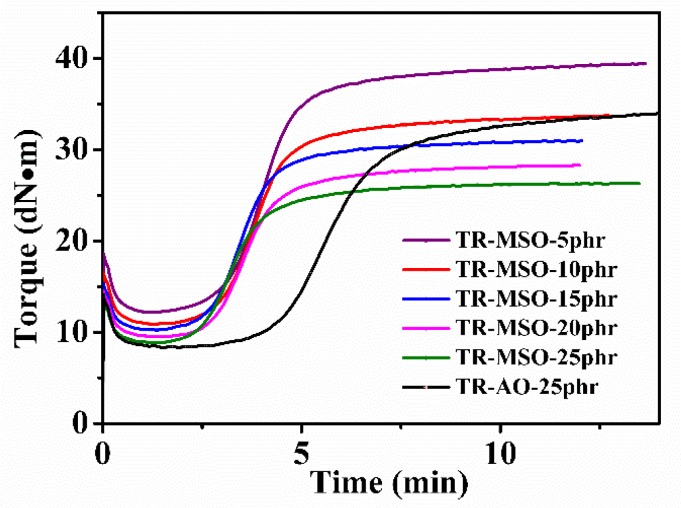
Vulcanization characteristics curve of tread rubber with different amounts of plasticizer.

The curing characteristics of the tire tread rubber plasticized with different quantities of MSO-6 is shown in Figure 6. With increasing contents of MSO-6S, both the maximum and minimum torques of the rubber are significantly reduced, which indicates that MSO-6S has very good plasticization effect. The decrease of minimum torque is in accord with the Mooney viscosity result.

The torque difference of TR-MSO is lower compared with that of TR-AO because MSO could also influence the crosslinking density. However, all the MSO-plasticized rubber has much shorter *T*_90_ than TR-AO, which could enhance the production efficiency and reduce the energy consumption during the rubber molding process.

#### 3.3.3. Mechanical Properties of Tread Rubber with Different Quantity of Plasticizer

**Figure 7:**
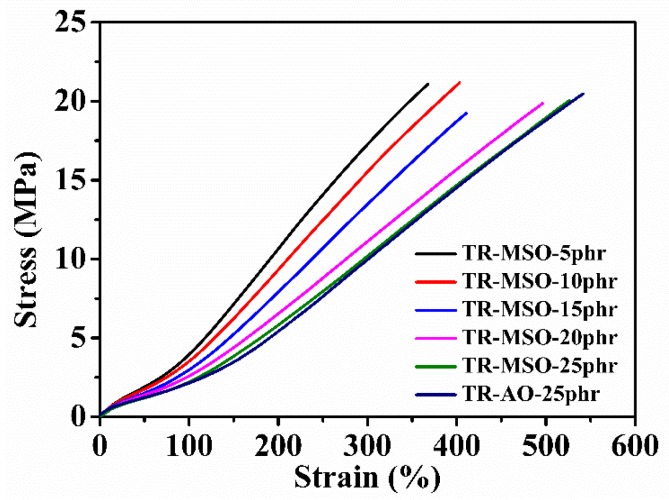
Stress-strain curves of tread rubber with different amounts of plasticizer.

The stress-strain curves of TR-MSO and TR-AO are shown in Figure 7 and the mechanical properties of TR-MSO and TR-AO are shown in Table 5. With increasing amount of MSO-6%, the hardness and modulus were reduced while the elongation at break increased due to the enhanced plasticization effect. Note that the tensile strength of TR-MSO is similar compared with TR-AO, means that the crosslinking densities of these rubbers are adequate. That is to say, after being modified with sulfur, MSO would not harm the crosslinking density. With the same content, the tensile strength, modulus, and hardness of TR-MSO-25phr and TR-AO are almost the same. Note that the aging coefficient (100 °C for 48 h) of TR-MSO-25phr is higher and the Akron abrasion loss is much smaller, compared with those of TR-AO. The small Akron abrasion loss means excellent wear resistance and longer life of the tire, which is very important for tires. In a word, the mechanical properties of MSO-6%-plasticized tire tread rubber are better than or at least comparable with those of AO-plasticized rubber.

## 4. Conclusions

Modified soybean oil (MSO) was synthesized from soybean oil and sulfur, and the double bond amount decreased from 4.6 to 1.7 per molecule with increasing weight percentage of sulfur from 0 to 9 wt %. The plasticization effect of three MSOs with different quantities of sulfur on a tire tread rubber was then systematically studied in comparison with SO. The crosslinking density and modulus of TR-MSO were significantly improved. TR-MSO-6% was found to have the best comprehensive properties, thus, the plasticization effect of TR-MSO-6% with different quantities of MSO-6% on TR was further studied. With the increase quantity of MSO-6%, the Mooney viscosity, Payne effect, hardness and modulus were all decreased, indicating an improved plasticization effect. TR-MSO-25phr was also compared with TR-AO to determine the potential application of MSO in tire tread rubber. The vulcanization time of rubber plasticized with MSO was found to be efficiently shortened compared with that of SO- and AO-plasticized rubber, which is beneficial for production efficiency and energy consumption reduction during the rubber molding process. The mechanical properties of MSO-6% plasticized tire tread rubber are better than or at least comparable with those of AO-plasticized rubber. More importantly, the aging resistance properties and wear resistance properties of MSO-6% plasticized rubber are better than those of AO-plasticized rubber, which means longer tire life. The modified soybean oil was a promising plasticizer for tire tread rubber to replace petroleum plasticizers, which would make an important contribution to the tire industry.

## Figures and Tables

**Table 1:** Tread rubber formula.

Ingredient	Recipe 1 (phr)	Recipe 2 (phr)
SBR	20.0	20.0
NR	40.0	40.0
BR	40.0	40.0
Plasticizer	0, 25 Variable (SO, MSO-3S%, MSO-6%, MSO-9%)	MSO-6% Variable(5,10,15,20,25), AO(25)
Silica	35.0	35.0
Carbon black	40.0	40.0
TESPT	2.8	2.8
SA	2.0	2.0
Zinc oxide	2.5	2.5
Antioxidant 4020	1.5	1.5
Accelerator CZ	1.7	1.7
Sulfur	1.9	1.9

**Table 2:** GPC data of soybean oil and modified soybean oil with different amounts of reagents.

Sample	*M*_n_ (×10^3^ g/mol)	*M*_w_ (×10^3^ g/mol)	Area Percentage (%)
SO	1.07	1.24	100
MSO-3%	3.30	3.63	17.72
	1.10	1.30	82.28
MSO-6%	3.90	4.98	42.33
	1.12	1.33	57.67
MSO-9%	4.69	7.18	57.10
	1.12	1.34	42.90

**Table 3:** Vulcanization characteristic of tread rubber with different plasticizers.

Number	*M*_H_ (dNm)	*M*_L_ (dNm)	Δ*M* (dNm)	*T*_90_ (min:sec)	Crosslink Density (10^−4^mol/mL)
Reference sample	52.33	14.28	38.05	8:29	/
TR-SO	27.01	7.96	19.05	7:31	1.69
TR-MSO-3%	27.14	7.98	19.16	5:28	1.70
TR-MSO-6%	29.41	8.91	20.50	4:31	1.73
TR-MSO-9%	31.24	10.18	21.06	4:36	1.76

**Table 4:** Mechanical properties of tread rubber with different plasticizers.

Sample	Elongation at Break (%)	Modulus at 100% (MPa)	Modulus at 300% (MPa)	Tensile Stress (MPa)	Shore A Hardness
Reference sample	328 ± 17	3.3 ± 0.1	16.6 ± 0.2	17.2 ± 1.7	73
TR-SO	618 ± 31	1.6 ± 0.1	7.7 ± 0.1	20.0 ± 1.4	55
TR-MSO-3%	553 ± 27	2.0 ± 0.3	8.8 ± 0.1	19.5 ± 0.5	56
TR-MSO-6%	533 ± 12	2.1 ± 0.1	9.7 ± 0.2	20.0 ± 0.8	57
TR-MSO-9%	504 ± 14	2.1 ± 0.2	9.2 ± 0.2	18.7 ± 1.4	58

**Table 5:** Mechanical properties of tread rubber with different amounts of plasticizer.

Property	Sample					
	1# 5 phr	2# 10 phr	3# 15 phr	4# 20 phr	5# 25 phr	6# TR-AO
Elongation at Break (%)	371 ± 21	407 ± 13	411 ± 15	503 ± 22	533 ± 18	542 ± 21
Modulus at 100% (MPa)	3.6 ± 0.1	3.2 ± 0.3	2.8 ± 0.1	2.4 ± 0.2	2.1 ± 0.1	2.2 ± 0.1
Modulus at 300% (MPa)	15.8 ± 0.4	14.2 ± 0.1	12.7 ± 0.1	10.3 ± 0.2	9.7 ± 0.1	10.0 ± 0.3
Tensile Stress (MPa)	19.8 ± 0.6	19.7 ± 0.4	19.5 ± 1.1	19.6 ± 0.7	19.6 ± 0.7	20.4 ± 1.3
Shore A Hardness	69	67	63	62	58	59
Akron Abrasion Loss (cm3)	/	/	/	/	0.1502	0.1805
Aging Coefficient (%)	/	/	/	/	90.74	88.74
